# Morphology of pollen in *Ferula* genus (Apiaceae)

**DOI:** 10.3897/phytokeys.179.66312

**Published:** 2021-07-13

**Authors:** Birol Baser, Mehmet Sagıroglu, Gulden Dogan, Hayri Duman

**Affiliations:** 1 Faculty of Arts and Sciences, Department of Biology, Bitlis Eren University, Bitlis, Turkey Bitlis Eren University Bitlis Turkey; 2 Faculty of Arts and Sciences, Department of Biology, Sakarya University, Sakarya, Turkey Sakarya University Sakarya Turkey; 3 Science Faculty, Department of Biology, Firat University, Elazig, Turkey Firat University Elazig Turkey; 4 Science Faculty, Department of Biology, Gazi University, Ankara, Turkey Gazi University Ankara Turkey

**Keywords:** Apiaceae, *
Ferula
*, LM, pollen morphology, SEM, Turkey

## Abstract

In this study, the pollen morphology of all *Ferula* species distributed throughout the country of Turkey was studied with light and scanning electron microscopy for the first time. The aim is to identify the pollen morphological characteristics of 23 *Ferula* species. The pollen is radially symmetrical, isopolar and tricolporate in all examined species. Pollen grains are prolate and perprolate with the polar axis ranging from 22.28 to 40.47 µm and the equatorial axis from 13.70 to 18.73 µm. Their polar shapes are triangular, triangular to subtriangular and circular to subcircular. Several types of exine ornamentations have been observed on pollen through the use of scanning electron microscopy. The dendrogram constructed by using Average Linkage of the examined data revealed two main groups. It was determined that some pollen characteristics are more useful for classification than others. In particular, P, E, the ratio of P/E (pollen shape) and ornamentation in the polar and equatorial views are the most valuable variables for discrimination the *Ferula* species.

## Introduction

Apiaceae is one of the largest plant families in the world. Apiaceae comprise approximately 450 genera and 3700 species, chiefly in north temperate regions (Pimenov and Leonov 2004).

The largest centere of biodiversity for this family in Asian countries is Turkey, with about 160 endemic species included in 44 genera (Bilgili et al. 2016). These family members are of considerable economic importance as food, flavouring and ornamental plants.

The genus *Ferula* is a very important pharmaceutical plant of the Apiaceae family. There are different opinions about the taxonomy on the subfamily, tribe, genus and species of Apiaceae as the taxonomic system of Apiaceae is based on the typical umbrella anthotaxy and its fruit with specific secretory tube. *Ferula* is traditionally classified in the tribe Peucedaneae and six subgenera are recognised with in the genus (Pimenov and Leonov 1993; Korovin 1947). However, serological studies by Shneyer et al. (1995) indicated the distant position of *Ferula* from all other genera of traditionally delimited Paucedaneae. *Ferula* species are typically tall perennials or biennials with stout stems and finely divided leaves with inflated sheaths. Morphological charactereristics of the basal leaves and mature fruits are necessary for the accurate identification of the species (Kurzyna-Młynik et al. 2008).

The chemical constituents of plants in the genus *Ferula* (Umbelliferea) have been fairly thoroughly studied, with the most common compounds being sesquiterpenes and sesquiterpene coumarins. As such, many members of the genus have been used in China for the treatment of rheumatoid arthritis and stomach diseases. Modern pharmacological studies have established the anti-ulcerative, antibacterial, anti-inflammatory and immunopharmacological activities of this genus (Yang et al. 2006; Li et al. 2014).

Globally, the genus *Ferula* L. (Apiaceae) contains 180–185 species, with the most diversity found in Central and Southwest Asia (Pimenov and Leonov 2004). In Turkey, about 130 species can be found, of which approximately 100 are endemic (Korovin 1951; Chamberlain and Rechinger 1987). The first revision of *Ferula* in Turkey was prepared by Peşmen (Peşmen 1972), where18 species were recognised, one of which was incompletely known and nine of which were endemic (Sağıroğlu and Duman 2008). A comprehensive revision of Turkish *Ferula* has been undertaken by Sağıroğlu since 2005 and a large number of specimens have been collected from all over Turkey. Four new species have since been added to the Flora of Turkey and one was made a synonym (Duman and Sağıroğlu 2005; Sağıroğlu and Duman 2006; Sağıroğlu and Duman 2007; Sağıroğlu and Duman 2010; Sağıroğlu and Duman 2014). *F. divaricata* and *F. pisidica* were published by Pimenov and Akalın (Pimenov and Kljuykov 2013; Akalın et al. 2020). According to recent studies, the *Ferula* genus includes 23 species, of which 14 species are endemic (Güner et al. 2012).

Pollen morphology of various members of the family Umbelliferae has been studied over time. For example, Erdtman (1952) studied pollen morphology of the family Umbelliferae, while Ting (1961) examined pollen of some American species of family Umbelliferae. Additionally, Ting et al. (1964) examined pollen morphology of the subfamily Hydrocotyloideae: Umbelliferae. Pollen morphology of the North European Flora of the family Umbelliferae was examined by Punt (1984). However, the most comprehensive study on pollen morphology of family Umbelliferae is that of Cerceau-Larrival (1971, 1981). Van Zeist et al. (1977) described the following pollen types from Iran: *Anisosciadium*, *Bunium*, *Eryngium*, *Ferula*, *Malabaila*, *Pimpinella*, *Sium erectum*, and *Turgenia* types. Pollen morphology of the family has also been studied by Erdtman et al. 1961, 1969; Visset 1972; Faegri and Iversen 1975; Nilsson et al. 1977; Moore and Webb 1978; Nigaud 1977, 1980; Punt 1984; Perveen and Qaiser 2006. Descriptions by several authors have been given in regional pollen floras (Aytuğ et al. 1971; Kuprianova and Alyshina 1972; Punt 1984; Perveen and Qaiser 2006; Punt et al. 2007; Güner et al. 2011; Pehlivan et al. 2009; Mungan et al. 2011; Baldemir et al. 2018), but only a few studies have been conducted on the genus *Ferula*.

To date, no information is available on the pollen morphology of species of *Ferula* found in Turkey. In the present study, an attempt has been made to provide complete information on pollen morphology of these genera in Turkey. For all 23 taxa belonging to the genus *Ferula*, pollen morphology was examined from samples which were collected from their natural habitat. This research is a palynological study of *Ferula*, collected from different regions in Turkey and was conducted to shed light on the properties of the pollen taxa that were examined. The present research aims to provide detailed quantitative and qualitative data on the pollen morphology of the genus, as well as to evaluate the taxonomic value of those data.

## Materials and methods

### Plant material

The material used for this study was collected from various locations throughout Turkey during the year 2018. The voucher specimens were deposited in the herbarium of Sakarya University and the Faculty of Science of Gazi University, Ankara, Turkey (GAZI). Plant localities, collection dates and collector numbers can all be seen in Table 1.

**Table 1.** List of taxa examined, localities and collector.

Taxa	Locality	Collector
*F. szowitsiana* D.C.	B6 Sivas: Zara road, 13. km, 1300 m, 14.06.2018	M.S 6859
*F. drudeana* Korovin	C5 Kayseri: Yahyalı, Faraşa Village, 1550 m, 10.06.2018	M.S 6854
*F. coskunii* H. Duman & M. Sağıroğlu	C6 Hatay: Hatay-Yayla Mountain, 1200 m, 15.07.2018	M.S 6881
*F. mervynii* M.Sağıroğlu & H. Duman	A9 Erzurum: Uzundere-Artvin road, Dam vicinity 27.07.2018	M.S 6885
*F. communis* L.	A6 Samsun: Samsun-Ankara road, Samsun output, 50 m, 03.05.2018	M.S 6829
*F. tingitana* L.	C1 Izmir: Efes ruins, 18.05.2018	M.S 6833
*F. duranii* M.Sağıroğlu & H. Duman	C3 Antalya: Alanya castle, 40 m, 30.05.2018	M.S 6844
*F. lycia* Boiss.	B4 Konya: Hadim-Bozkır road, 54. km, 1050 m, 29.05.2018	M.S 6842
*F. hermonis* Boiss.	C6 Adana: Between Gürümze-Feke, 1600 m, 12.06.2018	M.S 6857
*F. anatolica* Boiss.	B2 Manisa: Alaşehir, Kozluca Village, 1000 m, 25.05.2018	M.S 6836
*F. orientalis* L.	B7 Elazig: Elazig-Diyarbakir road, 62. km, 1250 m, 17.06.2018	M.S 6869
*F. brevipedicellata* Peşmen ex M. Sağıroğlu & H. Duman	B9 Bitlis: Hizan-Pervari road, 26. km, 1000 m, 15.06.2018	M.S 6861
*F. halophila* Peşmen	B4: Tuz Lake, islands, 908 m, 02.06.2018	M.S 6846
*F. parva* Freyn & Bornm.	C4 Konya: Karaman-Mut road, 5. km, 1130 m, 16.07.2018	M.S 3175
*F. tenuissima* Hub.-Mor. & Peşmen	C6 Osmaniye: Zorkun Plateau, 5. km, 1600 m, 15.07.2018	M.S 6880
*F. haussknechtii* Wolff ex Rech.	B9 Bitlis: Between Tatvan-Van, 66. km, 1950 m, 15.06.2018	M.S 6862
*F. elaeochytris* Korovin	C5: Nigde-Ulukışla, Alihoca-Maden villages between, 1500 m, 04.06.2018	M.S 6850
*F. longipedunculata* Peşmen	B6 Maraş: Maraş-Göksün, Keklikoluk Village, Işık Mountain, 1900 m, 18.06.2018	M.S 6872
*F. divaricata* Pimenov	B3: Eskişehir-Sivrihisar yolu, Beylikova road, 920 m, 19.06.2018	M.S 4542
*F. huber-morathii* Peşmen	B8 Bingöl: Elazig-Bingöl road, Yolçatı, 1300 m, 16.06.2018	M.S 6864
*F. caspica* Bieb.	A4Ankara: Ankara-Nallıhan, Davutoğlan, 500 m, 27.05.2018	M.S 6839
*F. rigidula* DC.	B5 Yozgat: Yozgat-Şefaatli output, 2. km, rocks, 920 m, 20.05.2018	M.S 6835
*F. pisidica* Akalın & Miski	C4 Konya: Hadim-Beyreli Village, 1570 m, 2 1.06.2018	M.S 6876

### Palynological and morphological analysis

**For Light Microscope Studies**: Pollen slides were prepared using the Wodehouse (1935) technique. The pollen grains were mounted in unstained glycerine jelly, stained with safranin and studies were made using an Olympus BX-21. The measurements were based on 30 readings from each specimen. Polar axis (P), equatorial diameter (E), P/E ratio, exine (ex), intine (in), colpi long axis (clg), colpi short axis (clt), pori long axis (plg), pori short axis (plt) and costae (c) were also measured.

**For SEM studies**: Dried pollen grains were transferred on to aluminium stubs and coated with gold at 20 Kv for 4 min in a sputter-coater. The SEM examination was carried using a ZEISS Supra 55 Scanning Electron Microscope at the SEM Laboratory of the Central Research Laboratory (MERLAB), Yuzuncu Yil University, Van.

The pollen terminology was adopted from Faegri and Iversen (1975), Punt (1984) and Punt et al. (2007) and the shape classification followed that of Erdtman (1969), based on the P/E ratio in Tables 2, 3.

**Table 2.** Pollen morphological parameters in the investigated taxa.

Taxon	P	E	P/E	Clg	Clt	Plg	Plt	Exine	Intine	Costa
*F. szowitsiana*	35.03±2.67	15.82±1.17	2.21	23.28±2.14	0.68±0.18	5.80±0.68	8.42±1.24	1.23±0.24	0.53±0.17	0.94±0.21
*F. drudeana*	34.27±2.13	15.18±1.42	2.26	23.23±2.47	0.63±0.17	5.73±0.64	7.07±0.81	1.38±0.14	0.48±0.14	1.03±0.19
*F. coskunii*	25.33±1.95	13.70±1.16	1.85	18.95±2.47	0.62±0.25	5.18±0.77	7.80±1.21	0.81±0.19	0.45±0.19	0.75±0.21
*F. mervynii*	22.28±2.17	14.40±1.50	1.55	16.83±1.90	0.70±0.20	3.77±0.68	4.52±0.88	0.79±0.27	0.51±0.17	0.68±0.17
*F. communis*	32.73±1.89	18.73±1.63	1.75	24.63±2.28	0.66±0.24	5.68±0.55	9.28±0.61	1.55±0.24	0.58±0.12	1.54±0.19
*F. tingitana*	32.03±1.59	15.27±1.08	1.78	27.27±1.34	0.81±0.22	5.87±0.64	7.00±0.71	0.98±0.16	0.39±0.13	1.24±0.20
*F. duranii*	30.73±2.03	18.72±1.43	1.64	24.27±1.41	0.45±0.18	5.20±0.92	7.08±0.79	0.68±0.21	0.37±0.10	0.71±0.12
*F. lycia*	34.43±1.37	16.33±1.21	2.11	25.92±1.66	0.72±0.17	5.35±0.45	6.73±0.67	1.40±0.27	0.65±0.26	1.30±0.27
*F. hermonis*	34.40±2.81	16.52±1.38	2.08	23.98±1.20	0.60±0.12	5.65±0.49	8.78±1.25	1.18±0.25	0.53±0.14	0.77±0.18
*F. anatolica*	27.83±1.34	16.47±1.28	1.69	27.17±1.68	0.78±0.19	5.07±0.76	6.87±0.92	1.09±0.12	0.50±0.18	0.73±0.15
*F. orientalis*	37.17±2.70	18.33±1.79	2.03	26.03±2.38	0.66±0.19	5.48±0.59	8.17±0.95	1.34±0.22	0.33±0.12	1.35±0.22
*F. brevipedicellata*	32.77±2.17	16.05±1.32	2.04	26.28±1.97	0.61±0.13	4.67±0.67	6.10±0.64	1.30±0.28	0.48±0.11	1.16±0.26
*F. halophila*	33.50±1.43	17.16±1.26	1.95	24.87±1.78	0.70±0.19	5.40±0.66	7.90±1.15	1.89±0.24	0.73±0.20	1.73±0.22
*F. parva*	33.15±2.19	16.33±1.20	2.03	24.03±2.65	0.50±0.11	5.03±0.94	6.30±1.20	1.68±0.23	0.47±0.18	1.53±0.19
*F. tenuissima*	40.47±3.09	17.23±1.68	2.35	30.73±2.65	0.78±0.21	5.70±0.48	8.80±0.68	1.40±0.25	0.39±0.213	1.37±0.16
*F. haussknechtii*	31.23±1.63	16.43±1.54	1.90	23.97±1.63	0.55±0.10	5.63±0.57	6.83±0.70	1.45±0.17	0.55±0.15	1.22±0.29
*F. elaeochytris*	30.83±1.70	16.10±1.18	1.92	25.17±1.62	0.48±0.16	3.91±0.62	5.50±1.03	1.23±0.21	0.42±0.18	1.52±0.22
*F. longipedunculata*	32.87±2.58	17.27±1.33	1.90	24.83±1.62	0.78±0.18	5.82±0.50	8.47±0.87	1.51±0.19	0.34±0.12	1.45±0.19
*F. divaricata*	34.70±2.60	17.20±1.42	2.02	24.77±1.77	1.02±0.21	5.65±0.53	8.20±0.82	1.52±0.18	0.58±0.25	1.50±0.19
*F. huber-morathii*	35.08±1.74	16.87±1.50	2.08	23.70±2.22	0.76±0.18	5.88±0.60	7.82±1.13	1.48±0.25	0.46±0.14	1.29±0.23
*F. caspica*	27.03±1.87	14.37±1.22	1.88	22.63±1.61	0.50±0.14	4.53±0.92	5.77±0.84	1.17±0.22	0.49±0.17	1.10±0.18
*F. rigidula*	33.90±1.88	18.20±1.61	1.86	19.83±1.97	0.53±0.16	6.05±0.56	5.95±0.58	1.55±0.27	0.48±0.16	1.67±0.21
*F. pisidica*	32.17±2.52	17.83±2.12	1.80	25.50±2.75	0.57±0.18	5.05±1.03	7.62±1.39	1.52±0.19	0.37±0.13	1.50±0.19

NOMENCLATURE: P: Polar axis E: Equatorial axis Clg: Length of the colpus Clt: Width of the colpus Plg: Length of the porus Plt: Width of the porus. (Average μm± standard deviation)

### Numerical analysis

Using the SPSS 21.0 statistical programme, clustering analysis was performed to determine the similarities between groups (Fig. 5). Seven palynological characters were selected to distinguish the 23 taxa of the genera (Table 3). Each of these palynological characters is included in the analysis by giving numerical values. Since Gower’s formula (Gower 1971) was modified by Podani (1999), it now allows the inclusion of ordinal variables and missing scores in the data matrix. Thus, it was used to calculate the primary mixed data for dissimilarities. Additionally, Scatterplot analysis was performed by using P and E values. The graph obtained is shown in Figure 6.

**Table 3.** Eight palynological characters to distinguish the 23 taxa of the genus *Ferula.*

No	Taxa	P	E	P/E	Exine	Costae	Ornamentation, polar area	Ornamentation, equatorial area	Ornamentation, pore around
**F1**	*F. szowitsiana*	35.03	15.82	Perprolate	1.23	0.94	striate-reticulate	rugulate	rugulate-striate
**F2**	*F. drudeana*	34.27	15.18	Perprolate	1.38	1.03	striate-reticulate	rugulate	rugulate-striate
**F3**	*F. coskunii*	25.33	13.70	Prolate	0.81	0.75	striate-perforate	rugulate	rugulate-striate
**F4**	*F. mervynii*	22.28	14.40	Prolate	0.79	0.68	striate-reticulate	rugulate-striate	rugulate-striate
**F5**	*F. communis*	32.73	18.73	Prolate	1.55	1.54	striate-reticulate	rugulate-perforate	striate-reticulate
**F6**	*F. tingitana*	32.03	15.27	Prolate	0.98	1.24	striate-reticulate	rugulate-striate	rugulate-striate
**F7**	*F. duranii*	30.73	18.72	Prolate	0.68	0.71	striate-reticulate	rugulate	striate
**F8**	*F. lycia*	34.43	16.33	Perprolate	1.40	1.30	rugulate-striate	rugulate-striate	rugulate
**F9**	*F. hermonis*	34.40	16.52	Perprolate	1.18	0.77	striate-reticulate	rugulate	striate
**F10**	*F. anatolica*	27.83	16.47	Prolate	1.09	0.73	striate-reticulate	rugulate	striate
**F11**	*F. orientalis*	37.17	18.33	Perprolate	1.34	1.35	striate-reticulate	rugulate	striate
**F12**	*F. brevipedicellata*	32.77	16.05	Perprolate	1.30	1.16	rugulate-striate	rugulate	rugulate-striate
**F13**	*F. halophila*	33.50	17.16	Prolate	1.89	1.73	striate-reticulate	rugulate	striate
**F14**	*F. parva*	33.15	16.33	Perprolate	1.68	1.53	striate-reticulate	rugulate	striate
**F15**	*F. tenuissima*	40.47	17.23	Perprolate	1.40	1.37	striate-reticulate	rugulate	striate
**F16**	*F. haussknechtii*	31.23	16.43	Prolate	1.45	1.22	rugulate-verrucate	verrucate	verrucate
**F17**	*F. elaeochytris*	30.83	16.10	Prolate	1.23	1.52	striate-reticulate	rugulate	rugulate-striate
**F18**	*F. longipedunculata*	32.87	17.27	Prolate	1.51	1.45	striate-reticulate	rugulate	striate
**F19**	*F. divaricata*	34.70	17.20	Perprolate	1.52	1.50	striate-reticulate	rugulate	rugulate-striate
**F20**	*F. huber-morathii*	35.08	16.87	Perprolate	1.48	1.29	striate-reticulate	rugulate	striate
**F21**	*F. caspica*	27.03	14.37	Prolate	1.17	1.10	striate-reticulate	rugulate	striate-reticulate
**F22**	*F. rigidula*	33.90	18.20	Prolate	1.55	1.67	striate-reticulate	rugulate	verrucate
**F23**	*F. pisidica*	32.17	17.83	Prolate	1.52	1.50	striate-reticulate	rugulate	striate

## Results

**Figure 1.**
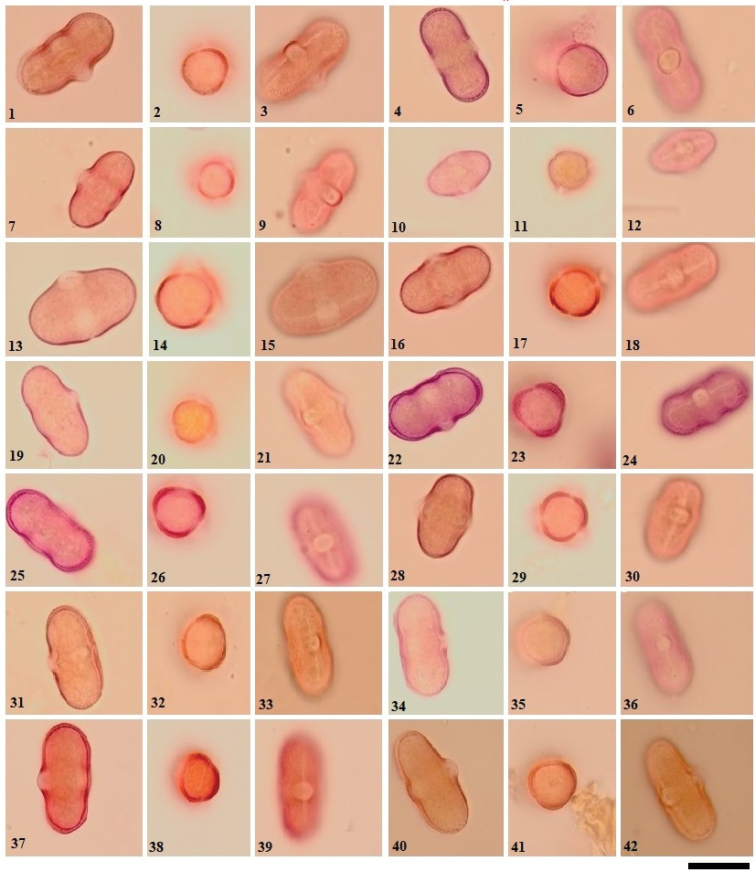
LM micrographs of pollen grains in the *Ferula* taxa examined **1–3***F. szowitsiana***4–6***F. drudeana***7–9***F. coskuni***10–12***F. mervynii***13–15***F. communis***16–18***F. tingitana***19–21***F. duranii***22–24***F. lycia***25–27***F. hermonis***28–30***F. anatolica***31–33***F. orientalis***34–36***F. brevipedicellata***37–39***F. halophila***40–42***F. parva.* Scale bar: 20 µm. (Equatorial view: **1, 4, 7, 10, 13, 16, 19, 22, 25, 28, 31, 34, 37, 40. Polar view: 2, 5, 8, 11, 14, 17, 20, 23, 26, 29, 32, 35, 38, 41**. Aperture view: **3, 6, 9, 12, 15, 18, 21, 24, 27, 30, 33, 36, 39, 42**).

**Figure 2.**
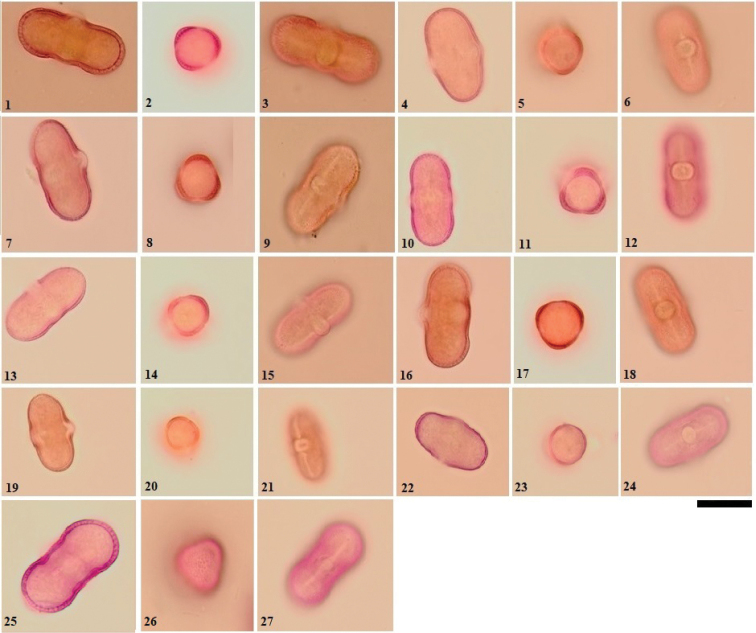
LM micrographs of pollen grains in the *Ferula* taxa examined **1–3***F. tenuissima***4–6***F. haussknechtii***7–9***F. elaeochytris***10–12***F. longipedunculata***13–15***F. divaricata***16–18***F. huber-morathii***19–21***F. caspica***22–24***F. rigidula***25–27***F. pisidica*. Scale bar: 20 µm. (Equatorial view: **1, 4, 7, 10, 13, 16, 19, 22, 25**. Polar view: **2, 5, 8, 11, 14, 17, 20, 23, 26**. Aperture view: **3, 6, 9, 12, 15, 18, 21, 24, 27**).

**Figure 3.**
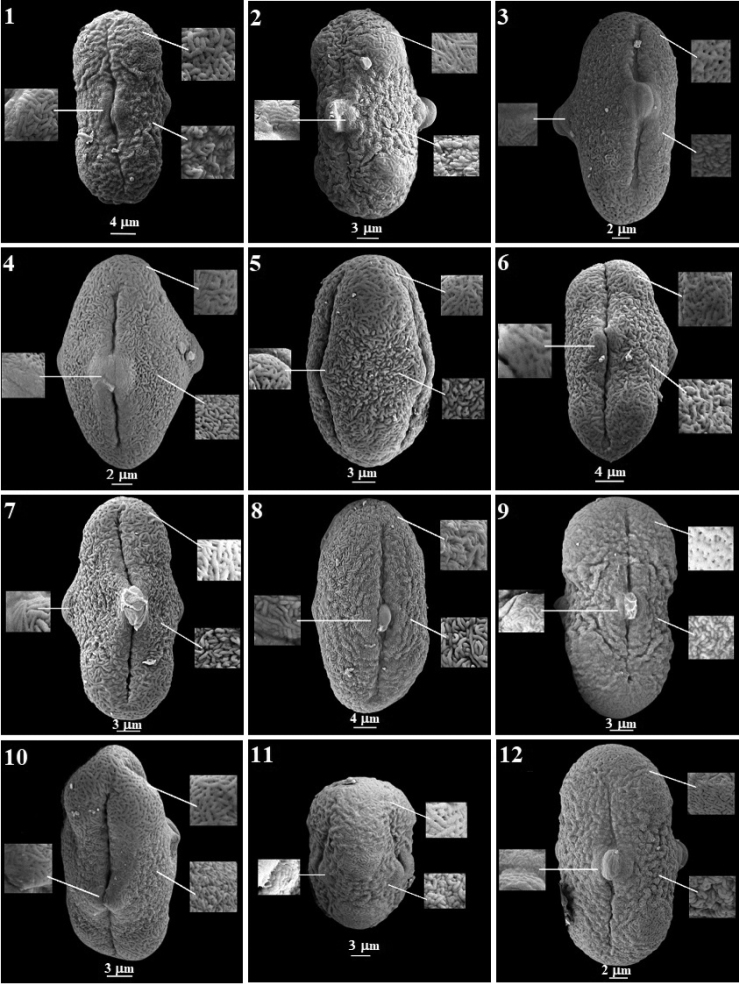
SEM micrographs of pollen grains in the *Ferula* taxa examined **1***F. szowitsiana***2***F. drudeana***3***F. coskuni***4***F. mervynii***5***F. communis***6***F. tingitana***7***F. duranii***8***F. lycia***9***F. hermonis***10***F. anatolica***11***F. orientalis***12***F. brevipedicellata*.

**Figure 4.**
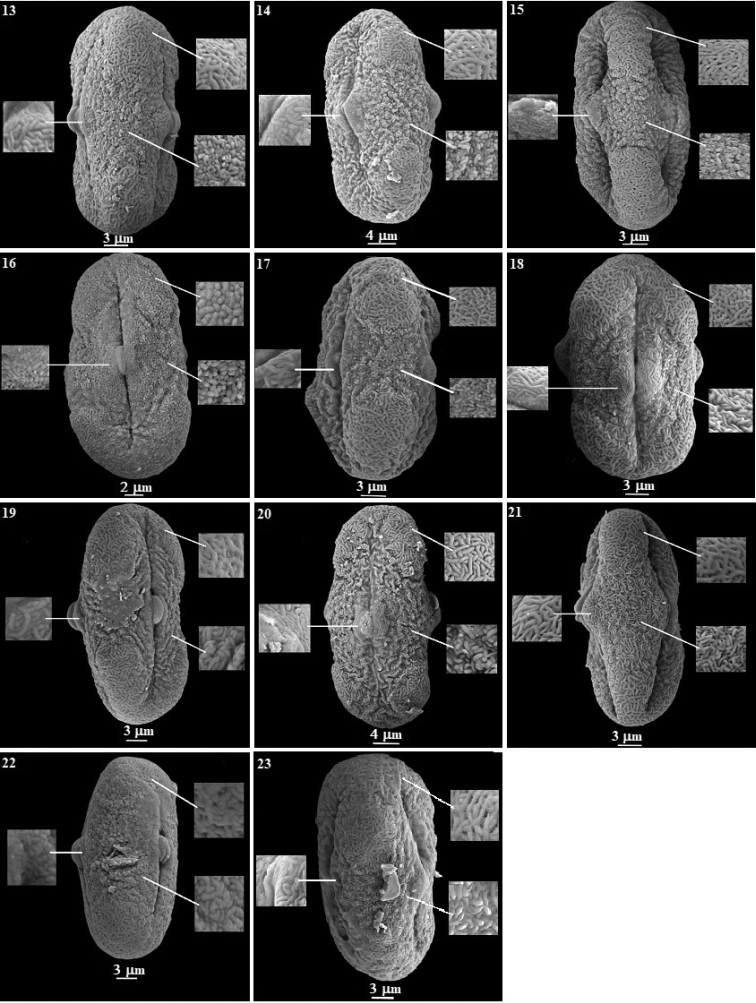
SEM micrographs of pollen grains in the *Ferula* taxa examined **13***F. halophila***14***F. parva***15***F. tenuissima***16***F. haussknechtii***17***F. elaeochytris***18***F. longipedunculata***19***F. divaricata***20***F. huber-morathii***21***F. caspica***22***F. rigidula***23***F. pisidica*.

**Figure 5.**
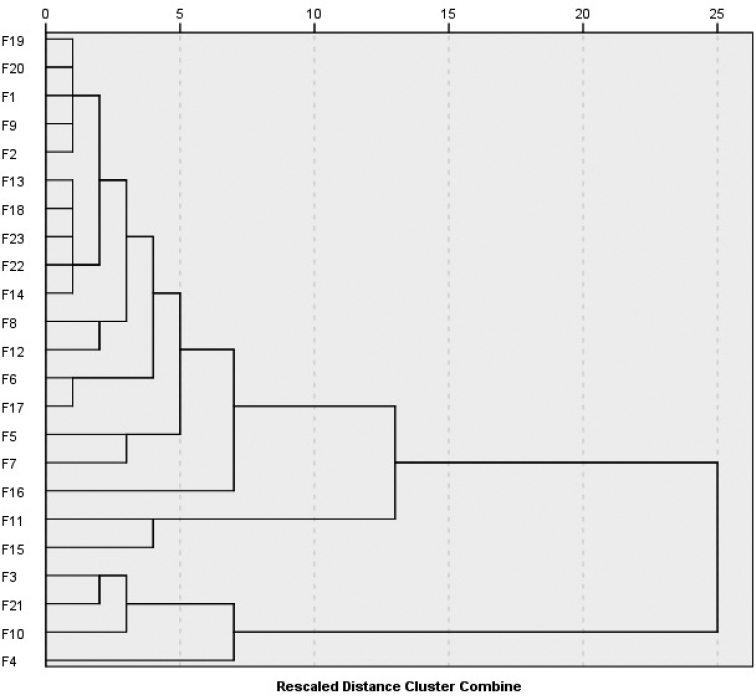
Dendrogram using Average Linkage (Between Groups) of the examined data (F1: *F. szowitsiana*; F2: *F. drudeana*; F3: *F. coskuni*; F4: *F. mervynii*; F5: *F. communis*; F6: *F. tingitana*; F7: *F. duranii*; F8: *F. lycia*; F9: *F. hermonis*; F10: *F. anatolica*; F11: *F. orientalis*; F12: *F. brevipedicellata*; F13: *F. halophila*; F14: *F. parva*; F15: *F. tenuissima*; F16: *F. haussknechtii*; F17: *F. elaeochytris*; F18: *F. longipedunculata*; F19: *F. divaricata*; F20: *F. huber-morathii*; F21: *F. caspica*; F22: *F. rigidula*; F23: *F. pisidica*).

**Figure 6.**
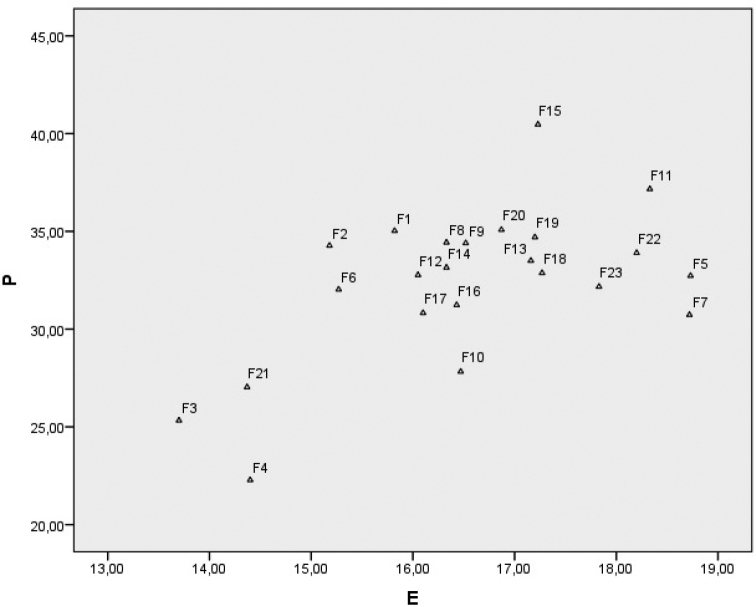
Graphs of Scatterplot (F1: *F. szowitsiana*; F2: *F. drudeana*; F3: *F. coskuni*; F4: *F. mervynii*; F5: *F. communis*; F6: *F. tingitana*; F7: *F. duranii*; F8: *F. lycia*; F9: *F. hermonis*; F10: *F. anatolica*; F11: *F. orientalis*; F12: *F. brevipedicellata*; F13: *F. halophila*; F14: *F. parva*; F15: *F. tenuissima*; F16: *F. haussknechtii*; F17: *F. elaeochytris*; F18: *F. longipedunculata*; F19: *F. divaricata*; F20: *F. huber-morathii*; F21: *F. caspica*; F22: *F. rigidula*; F23: *F. pisidica*).

The pollen properties of 23 species of Turkish *Ferula* are here described for the first time. All of the morphological parameters investigated are shown in Tables 2, 3 and in Figs 1–6. The pollen grains are radially symmetrical and isopolar. The shape is prolate and perprolate. Their apertures are operculate and tricolporate with costae. In this study, the genus *Ferula* was found to have three types of polar shapes; triangular, triangular to subtriangular, and circular to subcircular (Figs 1, 2).

The polar axis (P) ranges from 22.28 to 40.47 μm and the equatorial axis (E) ranges from 13.70 to 18.73 μm. The polar axis is longest in *F. tenuissima* (40.47 μm) and shortest in *F. mervynii* (22.28 μm); the equatorial axis is longest in *F. communis* (18.73 μm) and shortest in *F. coskunii* (13.70 μm). The dimensions are smaller in *F. mervynii* and larger in *F. tenuissima*. In all taxa examined, the width of the porus (plt) is greater than the width of the colpus (clt). The colpus is short to rather long (16.83–30.73 µm), narrow (0.45–1.02 µm) and slit-like. The highest values were observed in *F. tenuissima* and *F. tingitana*. *F. mervynii* has the smallest measures of colpus. Intine thickness ranges between 0.33 and 0.73 µm. Intine was thickest in *F. halophila* and thinnest in *F. orientalis.* The exine is tectate and 0.68–1.89 µm in thickness in the equatorial area. Exine was thickest in *F. halophila* and thinnest in *F. duranii*. There is a thickening around the aperture of exine (costae) with a decreasing diameter towards the poles. In addition, *F. halophila* (1.73 μm) and *F. rigidula* (1.1.67 μm) have the thickest costae (Table 2).

According to LM investigations; prolate pollen shape were observed in *F. coskunii*, *F. mervynii*, *F. communis*, *F. tingitana*, *F. duranii*, *F. anatolica*, *F. halophila*, *F. haussknechtii*, *F. elaeochytris*, *F. longipedunculata*, *F. caspica*, *F. rigidula* and *F. pisidica*. Other taxa are perprolate. In other words, in terms of pollen shape, about half of the studied taxa is prolate and the other half is perprolate.

Through SEM investigation, several types of ornamentations were observed in the equatorial area, polar area and around the pore on pollen surfaces. In many pollens, it has been determined that the ornamentation around the pore is different from that in both polar and equatorial areas. In the equatorial area, ornamentation was determined to be; rugulate in 18 species, rugulate-striate in three species, rugulate-perforate in one species and verrucate in one species. In the polar area, ornamentation was striate-reticulate in 19 species, rugulate-striate in two species, striate-perforate in one species, and rugulate-verrucate in one species. Around the pore area, ornamentation was; striate in 10 species, rugulate-striate in eight species, striate-reticulate in two species, verrucate in two species and rugulate in one species (Figs 3, 4).

In the genus *Ferula*, different palynological characters, which became evident over the course of the investigation, were measured, leading to the realisation of a dendrogram. This dendrogram shows the similarities or dissimilarities which exist amongst the taxa being studied. The dendrogram obtained in this research is based on the seven palynological variables of the 23 taxa of *Ferula* genus and is presented in Figure 5. The dendrogram was constructed by using Average Linkage (Between Groups) of the examined data revealed through two main groups. From the dendrogram, it is evident that *F. coskunii*, *F. mervynii*, *F. anatolica* and *F. caspica* are quite different from the other species and were the first to separate. In scatterplot graphs, it is seen that the species with the smallest (*F. mervynii*) and largest (*F. tenuissima*) pollen differ from other species (Fig. 6).

## Discussion

Pollen morphology of 23 taxa has been examined by light and scanning microscope. Pollen grains of *Ferula* taxa were generally tricolporate, with the shape of the grains being prolate and perprolate. The P/E ratio ranged from 1.55 to 2.35. The pollen in approximately half of the species examined in this study has a perprolate shape, which is characteristic for the Apiaceae family (Baczyńskı et al. 2021). In the analysis, according to the ratio of P and E values, the largest pollen grains were found in *F. tenuissima* and the smallest in *F. duranii* (Table 2).

The pollen morphologies of the Turkish *Ferula* species have taxonomic significance. Variation was mainly observed in pollen shape and pollen sculpturing. The sculpturing of the pollen exine is useful for ascertaining relationships amongst species (Brochmann 1992). They differ in sculpturing from the equatorial area to the poles. In addition, different ornamentations around the pore were found (Table 3, Figs 3, 4).

Prolate, perprolate and tricolporate pollen with costae grains were seen in all species. The common aperture type of Apiaceae pollen grains is 3-colporate (rarely 4-colporate porate) and colpi with costae (Perveen and Qaiser 2006; Yousefzadi et al. 2006; Pehlivan et al. 2009; Güner et al. 2011). *Ferula* pollen grains have only a tricolporate aperture. It has been suggested that the outline of the endo-aperture is most important for the identification of Apiaceae (Cerceau-Larrival 1962; Punt 1984; Hebda 1985).

Palynological contributions on the family Umbelliferae are numerous, but often fragmentary and concern few species (Anefrod 1960; Erdtman 1971; Moore and Webb 1978; Punt 1984; Faegri and Iversen 1989). The most complete and interdisciplinary studies to date have been carried out by Cerceau-Larrival (1962, 1963, 1965, 1967, 1968, 1971, 1974, 1981), Cerceau-Larrival and Deroquet (1975) and De Leonardis et al. (2009), which not only allow correlation between the shape of fruits with the symmetry of radiosymmetric pollen, the pollen shape with the size of cotyledonous leaves and the value of the P/E ratio with the phenotype stability of the belonging tribe, but also further deepen the knowledge of the shape of the pollen grains, the variability of the sporodermic wall and the presence of columellar hypertrophy as adaptation to environmental conditions.

Cerceau-Larrival (1962) divided the pollen of Umbelliferae into five types based on P/E ratio: subrhomboidal (type1, P/E: 1–1.5), subcircular (type 2, P/E: 1–1.5), oval (type 3, P/E: 1.5–2), subrectangular (type 4, P/E: 2) and equatorially constricted (type 5, P/E: over 2). In the present study, all of the taxa examined belong to all of the pollen types as described by Cerceau-Larrival, the oval-type with a P/E ratio of 1.5–2 to the equatorially constricted type with a P/E ratio greater than 2. The diversification in the family Apiaceae of the phyletic series was subrhomboidal > subcircular > oval > subrectangular > equatorially constricted (Gruas-Cavagnetto and Cerceau-Larrival 1978), where a suboval or rectangular shape is a more advanced feature (De Leonardis et al. 2008).

Cerceau-Larrival (1959, 1962) and Punt (1984) observed that the polar view of Apiaceae was important for the differentiation of the pollen types. In this study, it was found that the genus *Ferula* had three types of polar shapes: triangular, triangular to subtriangular and circular to subcircular. Punt (1984) divided the family into 50 pollen types, based on the two outer contour shapes (outer contour of mesocolpium side straight or convex and concave or slightly concave). The outer contours of generally examined taxa are concave, slightly concave or straight, while those of *F. mervynii* and *F. communis* are convex, according to Punt’s classification.

Pollen ornamentation is one of the most significant characteristics that can be used to separate taxa (Pınar et al. 2009; Mačukanović-Jocić et al. 2017; Zhang et al. 2017). It was observed in the present research that *Ferula* was striate-reticulate, rugulate-striate, striate-perforate and rugulate-verrucate in polar view; rugulate, rugulate-striate, rugulate-perforate and verrucate in equatorial view; and rugulate-striate, striate-reticulate, rugulate, striate and verrucate around the pore in exine ornamentation. This situation reflects the variation between the species.

Pollen morphology of 50 species representing 27 genera of the family Umbelliferae from Pakistan was examined by Perveen and Qaiser (2006). They determined that the pollen grains of Umbelliferae were generally tricolporate, the shape of the grains varied from prolate-perprolate, and the P/E ratio ranged from 1.2 to 2.6. Tectum is uniformly striate to striate-rugulate. In their study, all the taxa examined belong to all the pollen types as identified by Cerceau-Larrival; that is, subrhomboidal type to equatorially constricted type. The present study has very similar results to these palynological properties. According to the findings of this current study; pollen grains are tricolporate, the shape of the pollens is prolate-perprolate, the P/E ratio ranges from 1.55 to 2.35 and all of the examined taxa belong to the oval to subrhomboidal type. Related to the tectum, three distinct pollen types are recognized viz., in Pakistan, *Bupleurum gilessii*-type, *Pleurospermum hookeri*-type, *Trachyspermum ammi*-type. The tectal surface of the *Pleurospermum hookeri* type is rugulate-striate, which was observed particularly around the polar, equatorial and pore areas of the taxa examined in the present study.

In another study, Güner et al. (2011) observed the following ornamentation types of *Seseli*: rugulate in the equatorial area, psilate at the poles; striate-reticulate at equator, rugulate at poles; rugulate at equator, striate at poles; and rugulate-granulate at equator, striate at poles. Ornamentation types such as rugulate, striate-reticulate and striate were also observed in *Ferula* pollens.

The results of the cluster analysis show that the examined members of *Ferula* that fall into two main groups coincide with pollen sizes (Fig. 5). According to the dendrogram using Average Linkage (Between Groups) analysis, based on pollen morphological data, each species was distinctly separated from each other. Pollen morphological characteristics, such as polar axis (P), equatorial axis (E), the ratio of P/E and ornamentation at the polar and equatorial view, are the most valuable variables for separating the *Ferula* species. In the scatterplot graphs, *F. coskunii*, *F. mervynii*, *F. anatolica* and *F. caspica* species were grouped together, just as in the dendrogram. These species were found as the external taxa separating from the other taxa at first in the dendrogram and the plot (Figs 5, 6). It was found that the P value of these four species is smaller than the other species. At the same time, three of these four species have lower E values than other species. In this case, it can be said that the P value is the primary valuable variable and E value had secondary importance for separating the species of *Ferula*.

According to Cluster analysis; *F. szowitsiana*, *F. drudeana*, *F. hermonis*, *F. divaricata* and *F. huber-morathii* species are in the same clade. The pollen shapes of these taxa are perprolate and ornamentation in the polar area is striate-reticulate, while in the equatorial area it is rugulate. *F. halophila*, *F. parva*, *F. longipedunculata*, *F. rigidula* and *F. pisidica* are found in the same clade and have P and E values that are very close to each other, with ornamentation in the polar area being striate-reticulate and rugulate in the equatorial area. *F. lycia* and *F. brevipedicellata* are more similar, the pollen shapes of these taxa are perprolate and ornamentation in the polar area is rugulate-striate. *F. tingitana* and *F. elaeochytris* species are in the same clade and their pollen shapes are prolate, with ornamentation in the polar area being striate-reticulate. *F. communis* and *F. duranii* species are similar, with perprolate pollen shapes, E values that are very close to each other and ornamentation in the polar area being striate-reticulate. *F. orientalis* and *F. tenuissima* species are more similar because the pollen shapes of these two taxa are perprolate, exine and costa values are very close to each other and ornamentation in the polar area is striate-reticulate, while it is rugulate in the equatorial area. The species *F. haussknechtii* differs from the species closest to it in that its ornamentation is rugulate-verrucate in the polar area and verrucate in the equatorial area.

Some *Ferula* species in the same clade are closely related to each other morphologically; for example, *F. szowitsiana* and *F. drudeana*; *F. coskunii* and *F. mervynii*; *F. halophila*, *F. parva* and *F. rigidula*. In other words, in some species, palynological data support the separation of taxa according to morphological characteristics.

## Conclusion

In conclusion, analysis of pollen grains of 23 *Ferula* species in Turkey by LM and SEM revealed that palynological characteristics are reliable criteria for explaining the relationships between these species. The results of the cluster analysis showed that the most important variables in order to separate the taxa of *Ferula* in this study are the P and E values, ratio of P/E (pollen shapes) and ornamentation in the polar and equatorial area. In other words, these particular pollen characteristics seem to have the potential for evaluation of infrageneric relationships in the genus *Ferula*.
